# MET异常非小细胞肺癌诊疗中国专家共识（2026年版）

**DOI:** 10.3779/j.issn.1009-3419.2026.102.13

**Published:** 2026-05-20

**Authors:** Jun CHEN, Baohui HAN, Yi HU, Jian HU, Yuankai SHI, 中国老年保健协会肺癌专业委员会

**Keywords:** 肺肿瘤, MET异常, *MET*ex14跳跃突变, *MET*扩增, MET蛋白过表达, 靶向治疗, MET-TKIs, Lung neoplasms, MET abnormalities, *MET* exon 14 skipping mutation, *MET* amplification, MET protein overexpression, Targeted therapy, MET-TKIs

## Abstract

间质上皮细胞转化因子（mesenchymal-epithelial transition factor, *MET*）基因位于人类第7号染色体，在细胞增殖、迁移、侵袭及血管新生等生理过程中发挥关键调节作用。作为非小细胞肺癌（non-small cell lung cancer, NSCLC）的主要驱动基因之一，MET异常可表现为*MET*基因14号外显子（*MET* exon 14, *MET*ex14）跳跃突变、基因拷贝数扩增、蛋白水平过表达、基因融合及活化突变等多种形式。本共识由中国老年保健协会肺癌专业委员会牵头，在2025版基础上进行了重要更新：提高*MET*扩增与蛋白过表达检测的推荐级别，要求对所有初诊及表皮生长因子受体-酪氨酸激酶抑制剂（epidermal growth factor receptor-tyrosine kinase inhibitors, EGFR-TKIs）耐药后的NSCLC患者常规开展这两项检测；规范了驱动基因阴性和EGFR-TKIs耐药后*MET*扩增的靶向治疗策略；针对MET蛋白过表达，分别就MET-TKIs耐药后与驱动基因阴性两种情境给出具体治疗建议，旨在为临床精准治疗提供更具操作性的参考。

## Abstract (en)

The mesenchymal-epithelial transition factor (*MET*) gene, located on human chromosome 7, exerts critical regulatory roles in cellular processes including proliferation, migration, invasion, and angiogenesis. As a key driver gene in non-small cell lung cancer (NSCLC), MET abnormalities encompass *MET* exon 14 (*MET*ex14) skipping mutations, gene amplification, protein overexpression, gene fusions, and activating mutations. This consensus, developed by the Lung Cancer Specialty Committee of the Chinese Elderly Health Care Association, updates the 2025 version with several key modifications: elevating the recommendation level for *MET* amplification and protein overexpression testing, advocating routine testing for all newly diagnosed NSCLC patients and those with acquired resistance to epidermal growth factor receptor-tyrosine kinase inhibitors (EGFR-TKIs); standardizing targeted therapy approaches for *MET* amplification in driver gene-negative settings and following EGFR-TKIs resistance; and subdividing MET protein overexpression-related management into post-EGFR-TKIs resistance and driver gene-negative categories with corresponding treatment protocols, thereby offering more actionable guidance for precise clinical decision-making.

间质上皮细胞转化因子（mesenchymal-epithelial transition factor, MET）作为非小细胞肺癌（non-small cell lung cancer, NSCLC）的重要肿瘤驱动基因，其临床价值日益凸显。随着检测技术的进步和新药的持续研发，MET异常NSCLC患者的诊疗水平不断提升，预后得到改善^[1]^。MET异常主要包括基因突变、基因扩增、蛋白过表达和基因融合等类型。其中MET 14号外显子（MET exon 14, METex14）跳跃突变和MET扩增在我国已有获批靶向药物，具有明确临床可操作性，成为NSCLC精准诊疗的重要靶点^[2]^。

为规范MET异常NSCLC的临床诊疗，中国老年保健协会肺癌专业委员会牵头制定并发布《MET异常NSCLC诊疗专家共识（2025版）》^[3]^，为临床实践提供了重要指导。鉴于MET异常相关检测技术与靶向药物研发更新迅速，最新研究证据不断积累，中国老年保健协会肺癌专业委员会在2025版共识基础上，系统梳理国内外最新循证证据与临床研究成果，经多学科专家充分研讨、审慎修订，形成《MET异常非小细胞肺癌诊疗中国专家共识（2026版）》，以期进一步指导临床规范化诊疗。

本共识专家组由全国多地区、多学科的肺癌诊疗专家组成。专家组严格遵循循证医学原则，系统检索国内外MET异常相关文献，筛选高证据等级、贴近临床实践的研究作为参考。经多轮专家论证，就MET异常检测与治疗的核心问题达成一致意见，最终形成本共识。

在整合专家临床经验的基础上，最终形成10项具有临床指导价值的共识。共识推荐强度分级如下：1A级：基于高质量荟萃分析或随机对照试验，专家意见高度一致；1B级：证据级别与1A级相同，但专家存在部分分歧；2A级：证据级别较低，但专家意见一致；2B级：证据级别较低，专家意见虽有分歧但差异不大；3级：专家组成员间存在显著争议。

本共识已在国际实践指南注册与透明化平台（http://guidelines-registry.org）注册，注册号：PREPARE-2026CN1316。

## 1 MET异常的概念和流行病学特点

### 1.1 MET基因

MET基因是一种编码蛋白酪氨酸激酶（tyrosine kinase, TK）的原癌基因，DNA序列全长约125 kb，其产物是肝细胞生长因子受体（hepatocyte growth factor receptor, c-Met/HGFR），该受体属于跨膜受体TK（receptor tyrosine kinase, RTK）家族。c-Met蛋白分为胞外、胞内两大功能区域，胞外区由SEMA、PSI及IPT1-4结构域构成，主要协同参与蛋白功能调控；胞内区包含近膜结构域、TK结构域（TK domain, TKD）及C端多功能对接位点（C-terminal multif unctional docking site, MDS）组成。

生理状态下，肝细胞生长因子（hepatocyte growth factor, HGF）与c-Met蛋白特异性结合后，可诱导受体二聚化与自身磷酸化，进而激活丝裂原活化蛋白激酶（mitogen-activated protein kinase, MAPK）、磷脂酰肌醇3-激酶/蛋白激酶B（phosphatidylinositol 3-kinase/protein kinase B, PI3K/Akt）、信号转导和转录活化因子3（signal transducer and activator of transcription 3, STAT3）等下游信号通路，调控细胞增殖、迁移、侵袭及血管生成等生物学过程。而MET基因发生异常改变时，会导致信号通路持续异常活化，驱动肿瘤发生、发展或诱导耐药。

MET基因异常主要为基因扩增和基因突变。MET扩增高发于肺癌、胃癌、乳腺癌等恶性肿瘤，通过上调c-Met蛋白表达持续激活致癌信号；MET基因突变整体发生率较低，但在NSCLC、肾细胞癌等特定肿瘤中已鉴定出多种MET基因突变亚型。此外，肿瘤微环境中肿瘤细胞与间质细胞的相互作用可诱发HGF过表达，进一步持续激活c-Met，形成致癌正反馈环路。深入解析MET异常的调控机制，对MET异常肿瘤患者的精准筛查、靶向治疗选择、疗效预测及预后改善具有重要的临床指导价值^[2]^。

### 1.2 METex14跳跃突变

METex14跳跃突变核心机制为mRNA剪接过程中缺失14号外显子编码序列^[4]^。这种结构变化阻碍了MET蛋白与泛素化蛋白的正常结合，使蛋白降解途径受阻，从而维持持续的TK信号转导活性，持续激活下游致癌信号，增强肿瘤细胞增殖、迁移、侵袭等恶性生物学特性。

该突变的发病率存在人群与病理亚型差异：中国NSCLC人群METex14跳跃突变检出率为0.9%-2.0%^[5-8]^，西方人群检出率略高，为2%-4%^[9]^。病理亚型层面，肺腺癌突变率约3%^[10]^，肺鳞癌为1%-2%^[11]^，而肺肉瘤样癌则显著升高至13%-22%^[12,13]^。该突变可与表皮生长因子受体（epidermal growth factor receptor, EGFR）异常共存，其中合并EGFR突变的比例为0.3%-10.0%，合并EGFR扩增的比例为6.4%-28.5%^[14]^。

流行病学特征显示，METex14跳跃突变好发于老年患者^[14]^，患者中位年龄为72.5岁^[15]^。此类突变肿瘤侵袭性强，易诱导多重抗肿瘤药物耐药，患者整体预后较差^[16]^。临床数据显示，该突变NSCLC患者接受一线化疗的中位总生存期（overall survival, OS）仅6.7个月^[16]^；一线免疫治疗疗效同样有限，局部晚期或转移性患者的客观缓解率（objective response rate, ORR）仅17%-35.7%，中位无进展生存期（progression-free survival, PFS）仅1.9-4.9个月^[17]^。究其原因，此类患者肿瘤突变负荷（tumor mutational burden, TMB）普遍偏低，即便程序性细胞死亡配体1（programmed cell death ligand 1, PD-L1）阳性甚至高表达，免疫单药治疗获益并不理想，且该特征在肺肉瘤样癌患者中更为突出^[18]^。目前，针对PD-L1阳性合并METex14跳跃突变的NSCLC患者，仍缺乏能带来显著临床获益的标准一线方案。

### 1.3 MET扩增

MET扩增是指肿瘤细胞中MET基因拷贝数异常增多，进而导致c-Met蛋白过度表达、通路持续激活，驱动肿瘤增殖、侵袭与转移，是NSCLC重要的致癌机制与耐药机制。根据基因变异形式，可分为局部扩增与7号染色体多倍体两类，其中局部扩增发生率为1%-7%，7号染色体多倍体为7%-23%，整体总发生率为10%-26%^[19]^，数据差异主要与检测方法、样本来源、入组人群有关。

MET扩增的致癌模式主要包含3种：一是作为靶向治疗后的获得性耐药事件，也是临床最常见模式；二是初治肿瘤患者中与EGFR等驱动基因突变共存，协同致癌；三是作为独立驱动因素诱发肿瘤。在耐药场景中，第一、二代EGFR-TKIs治疗耐药后，MET扩增发生率为5%-22%^[20-21]^；第三代EGFR-酪氨酸激酶抑制剂（tyrosine kinase inhibitors, TKIs）奥希替尼一线治疗耐药后，其发生率达30.9%^[22]^，二线治疗耐药后为5%-50%^[23]^。此外，MET扩增也是间变性淋巴瘤激酶（anaplastic lymphoma kinase, ALK）-TKIs的重要耐药机制，第二、三代ALK-TKIs治疗耐药后，其发生率约为13%^[24]^。

### 1.4 MET蛋白过表达

MET蛋白过表达独立于基因扩增与突变，是MET通路异常活化的重要形式，其发生机制复杂且受多因素调控。核心诱因包括：EGFR、Kirsten大鼠肉瘤病毒癌基因同源物（Kirsten rat sarcoma viral oncogene homolog, KRAS）等致癌基因异常激活，间接上调MET蛋白表达；肿瘤缺氧微环境激活缺氧诱导因子1α（hypoxia inducible factor-1α, HIF-1α）等转录因子，促进MET基因转录；同时，肿瘤微环境中的炎症因子、促血管生成因子及HGF信号异常、MET基因转录失调，均会进一步加剧MET蛋白过表达^[25]^。

中国NSCLC患者MET蛋白过表达发生率为17.5%-63.7%，西方人群为35%-72%^[26-28]^。经EGFR-TKIs治疗的EGFR突变晚期NSCLC患者，MET蛋白过表达率为30.4%-37.0%，EGFR野生型患者中发生率更高，达44.4%^[29]^；病理亚型中，肺腺癌患者可达65%^[30]^。

### 1.5 MET基因融合

MET基因可与多种基因发生融合变异，融合基因可驱动MET受体持续活化，激活下游致癌信号通路，促进肿瘤发生进展。该变异在NSCLC中整体发生率较低，其中KIF5B-MET为临床最常见的MET融合亚型^[31]^。

### 1.6 MET活化突变

除METex14跳跃突变外，MET突变可发生在TKD、胞外Semaphorin（SEMA）结构域或近膜结构域。目前在NSCLC患者中已证实的功能性突变包括胞外结构域E168D、N375S突变，可促进SEMA结构域二聚化；近膜结构域R988C、T1010I突变及D1020、Y1021位点氨基酸置换，也可异常激活MET信号通路。

## 2 MET异常的检测

### 2.1 检测MET异常的必要性

#### 2.1.1 国内外权威指南均推荐METex14跳跃突变为必检基因

美国国立综合癌症网络（National Comprehensive Cancer Network, NCCN）^[32]^、MET异常NSCLC诊疗专家共识（2025版）^[3]^、欧洲肿瘤内科学会（European Society for Medical Oncology, ESMO）^[33]^、IV期原发性肺癌中国治疗指南（2026版）^[34]^以及中国临床肿瘤协会（Chinese Society of Clinical Oncology, CSCO）^[35]^等国内外权威指南，均一致推荐将METex14跳跃突变列为必检基因。目前，全球已有多种针对METex14跳跃突变的MET-TKIs获得临床批准。对于晚期NSCLC患者而言，检测METex14跳跃突变有助于筛选出可能从MET-TKIs靶向治疗中获益的人群。除上述国际指南外，国内最新发布的中华医学会肺癌临床诊疗指南、亚洲胸部肿瘤学研究小组（Asian Thoracic Oncology Research Group, ATORG）制定的NSCLC MET改变专家共识^[36]^、中国专家团队达成的NSCLC MET检测临床共识^[37]^以及2026版中国IV期原发性肺癌诊疗指南^[34]^也均对此予以明确推荐。

#### 2.1.2 国内外权威指南均推荐进行MET扩增及过表达检测

MET基因扩增与MET蛋白过表达既是NSCLC的原发性致癌驱动因素，也是EGFR-TKIs继发性耐药关键机制之一，具备重要的临床检测与指导治疗价值。目前，MET-TKIs已获批用于MET扩增相关肿瘤的治疗，证实MET扩增可作为指导临床治疗的重要分子标志物。同时，美国食品药品监督管理局（Food and Drug Administration, FDA）已批准MET抗体偶联药物（antibody drug conjugate, ADC）的相关适应证，证实MET蛋白过表达可作为独立的生物标志物。晚期NSCLC患者尤其是EGFR-TKIs耐药后患者的后续治疗选择较为有限，多项临床研究证实，存在MET基因扩增^[38-40]^或MET蛋白过表达^[41,42]^的EGFR-TKIs耐药患者，接受MET-TKIs治疗可获得明确临床获益。基于循证医学证据，国内外多项权威肿瘤诊疗指南^[32,35-37,43]^均推荐将MET扩增与MET蛋白过表达纳入晚期NSCLC患者的检测项目。目前，EGFR-TKIs耐药后再次进行基因检测已成为临床常规操作，对于无法获取肿瘤组织标本的患者，液体活检可作为有效的补充检测手段，满足临床基因检测需求^[44]^。

专家共识1：推荐所有病理类型（包括腺癌、鳞癌、肉瘤样癌等）的晚期NSCLC患者在诊断时常规进行METex14跳跃突变（1A）、MET基因扩增（1A）及MET蛋白过表达（2B）检测；对于EGFR-TKIs耐药后的NSCLC患者，推荐进行MET基因扩增（1A）和MET蛋白过表达（2B）检测。

### 2.2 MET异常的检测方法

#### 2.2.1 METex14跳跃突变检测方法

实时定量聚合酶链式反应（real-time quantitative polymerase chain reaction, RT-qPCR）通过设计覆盖MET基因13和15号外显子区域的特异性引物，可高效检测RNA水平的突变情况。该方法操作简便、周期短、结果准确，应用广泛。需注意，该技术对Y1003位点突变可能存在检测盲区，且因RNA易降解，实验全程需严格把控样本质量，确保核酸完整性。

DNA下一代测序（next-generation sequencing, NGS）采用杂交捕获或扩增子建库策略，特异性富集METex14跳跃突变相关基因组区域以实现靶向测序。该技术通量高、准确性较好，但检测周期较长，且检测效能可能受引物优化程度、生物信息学分析水平及探针覆盖范围等多重因素制约。文库构建时需确保覆盖MET基因13号内含子、14号外显子及其下游50 bp区域。此外，用于NGS数据分析的参考数据库必须包含完整的METex14跳跃突变位点信息，并保持动态更新。

RNA NGS可通过RNA层面检测MET基因13、15号外显子的融合情况，为METex14跳跃突变的诊断提供直接依据。该方法检测范围明确、操作流程简便，但存在样本质量要求高、检测周期长、临床可及性有限等不足。与RT-qPCR类似，该方法对某些罕见MET变异类型也可能存在漏检风险。

专家共识2：METex14跳跃突变可采用RT-qPCR、DNA NGS或RNA NGS进行检测，各方法均有其优缺点，必要时可相互验证或补充检测（1A）。

#### 2.2.2 MET基因扩增检测方法

MET基因扩增可采用荧光原位杂交（fluorescence in situ hybridization, FISH）和DNA NGS两种方法检测。

FISH技术是检测MET基因扩增的金标准，其判定标准主要有两种。依据Cappuzzo标准，即通过每个细胞中MET基因拷贝数（gene copy number, GCN）进行定量分析。该方法操作简便、临床适用性强，但存在将多倍体误判为局部扩增的固有缺陷，可能导致结果偏差。美国科罗拉多大学癌症中心（University of Colorado Cancer Center, UCCC）提出的第二种方法采用MET基因与7号染色体着丝粒（centromere of chromosome 7, CEP7）的比值（MET/CEP7）进行计算，有效弥补了前一方法的不足。该方法以MET/CEP7≥2.0作为扩增判定标准，并将扩增程度细分为低、中、高三个等级，从而更精确地评估非多倍体情况下的MET扩增水平^[19]^，显著提升了检测准确性。研究数据显示，随着MET扩增水平提升，伴随其他基因变异的比例呈下降趋势，其驱动作用更为突出，对MET-TKIs类药物的治疗敏感性也相应增强。在识别真正MET扩增病例方面，Cappuzzo标准的表现逊于MET/CEP7比值法^[45]^。

DNA NGS可凭借测序深度及肿瘤细胞含量占比等信息，计算MET基因拷贝数变异。随着技术进步，DNA NGS与FISH检测的一致性有所提高。以国内两家NGS检测公司的产品为例，与FISH（GCN≥5或MET/CEP7≥2）检测的一致性分别达79.2%和77.6%^[46-47]^，阴性一致率分别为95.1%和89.0%，提示NGS检测MET扩增的假阳性率较低。NGS检测的拷贝数结果可通过公式转换为肿瘤细胞内的校正拷贝数，公式为：拷贝数=[200×差异倍数-2×（100-肿瘤纯度%）]/肿瘤纯度%^[48]^。相关检测产品应优化肿瘤占比的生信计算方法，报告校正后拷贝数，以更精确地指导治疗。

专家共识3：MET基因扩增可采用FISH和DNA NGS方法检测，FISH是检测的金标准（1A）；DNA NGS检测与FISH有较好一致性，但需进一步优化和验证，建议报告校正后的肿瘤细胞内MET拷贝数（2B）。

#### 2.2.3 MET蛋白过表达检测方法

免疫组织化学法（immunohistochemistry, IHC）是目前临床检测MET蛋白过表达的标准方法。其原理基于抗原-抗体特异性结合，通过酶、荧光素等标记抗体显色，对组织细胞内MET抗原进行定位、定性及相对定量分析。准确判读染色强度并评估肿瘤细胞阳性比例，是MET IHC检测的关键。目前，临床评分为公认的判读体系。SAVANNAH^[38]^、INSIGHT^[49]^、TATTON^[50]^等多项关键研究均探索性地采用该标准，即结合染色强度与阳性细胞比例综合评分，常规以≥50%肿瘤细胞呈中/强染色作为入组阈值；SCC244-104及SCC244-108两项研究的汇总分析也入组了≥50%肿瘤细胞强染色的患者^[51]^。2025年5月，美国FDA加速批准了首个靶向MET过表达的ADC药物Telisotuzumab vedotin（Teliso-V），并同步批准VENTANA MET（SP44）RxDx Assay作为其伴随诊断方法。该方法对MET蛋白表达的临床界值定义为在非鳞NSCLC中，≥50%的肿瘤细胞表现出强细胞膜和/或细胞浆染色（3+）^[52]^。

目前，国内外监管机构已批准SP44、c-MET、MET4等克隆号的MET抗体产品用于临床检测。尽管不同抗体的染色性能可能存在差异，但SP44克隆号作为首个获批的MET伴随诊断试剂，为筛选可能从Teliso-V治疗中获益的人群提供了明确依据。未来需进一步探究不同抗体平台间的一致性，探索其他潜在获益人群，推动MET IHC检测标准的统一与规范化。

专家共识4：推荐使用IHC进行MET蛋白过表达检测，建议采用循证医学证据充足的IHC检测平台、抗MET抗体及二抗检测试剂。结果判读建议参考“临床评分”标准，综合染色强度与肿瘤细胞阳性比例进行评分（2B）。

## 3 MET异常的治疗

### 3.1 METex14跳跃突变的治疗

目前，赛沃替尼、谷美替尼、伯瑞替尼、特泊替尼、卡马替尼等MET-TKIs均已获中国国家药品监督管理局（National Medical Products Administration, NMPA）批准上市（赛沃替尼2021年6月22日批准，谷美替尼2023年3月7日批准，伯瑞替尼2023年11月14日批准，特泊替尼2023年12月8日批准，卡马替尼2024年6月11日批准），并被多部权威指南与共识推荐，用于METex14跳跃突变NSCLC的治疗。临床研究数据^[53-59]^显示，此类药物用于初治患者ORR为62%-77%，经治患者为39%-70%，中位PFS为8.5-14.3个月，中位OS为19-28个月（表1）。

**表 1:** METex14跳跃突变靶向药物治疗局部晚期或转移性NSCLC的关键临床研究数据

药物名称	治疗线数	n	ORR （%，95%CI）	DCR （%，95%CI）	中位DoR （月，95%CI）	中位PFS （月，95%CI）	中位OS （月，95%CI）	主要不良反应 （发生率≥20%）
赛沃替尼^[53,54]^	一线	87	62.1（51.0-72.3）	92.0（84.1-96.7）	12.5（8.3-15.2）	13.7（8.5-16.6）	28.3（17.5-NE）	外周水肿、恶心、低白蛋白血症、ALT与AST升高、呕吐、食欲减退、乏力、低血钾、贫血、咳嗽
	二线及以上	79	39.2（28.4-50.9）	92.4（84.2-97.2）	11.1（6.6-NE）	11.0（8.3-16.6）	25.3（20.5-30.5）	
谷美替尼^[55,56]^	总人群	79	66（54-76）	84（74-91）	8.3（6.2-NE）	8.5（7.6-9.7）	19.4（12.1-30.1）	外周水肿、头痛、恶心、食欲减退、低白蛋白血症、ALT升高、呕吐
	一线	44	71（55-83）	89（74-91）	15.0（6.3-NE）	11.7（7.6-21.9）	25.4（11.7-NA）	
	二线及以上	35	60（42-76）	77（60-90）	8.2（5.1-NE）	7.6（4.1-9.6）	16.3（8.7-NA）	
伯瑞替尼^[57]^	总人群	52	75.0（61.1-86.0）	96.2（86.8-99.5）	16.5（9.2-19.4）	14.3（6.4-18.2）	20.3（16.2-29.7）	外周水肿、低白蛋白血症、低蛋白血症和贫血
	一线	35	77.1（59.9-89.6）	97.1（85.1-99.9）	17.1（9.2-21.8）	14.5（6.3-20.3）	20.3（16.2-NE）	
	二线及以上	17	70.6（44.0-89.7）	94.1（71.3-99.9）	15.3（3.7-19.4）	7.7（3.7-20.3）	20.7（13.7-NE）	
特泊替尼^[58]^	总人群	313	51.4（45.9-57.1）	76.0（70.9-80.7）	18.0（12.4-46.4)	11.2（9.5-13.8）	19.6（16.2-22.9）	外周水肿、低白蛋白血症、恶心、腹泻和血肌酐水平升高
	一线	164	57.3（49.4-65.0）	78.7（71.6-84.7）	46.4（13.8-NE）	12.6（9.7-17.7）	21.3（14.2-25.9）	
	二线及以上	149	45.0（36.8-53.3）	73.8（66.0-80.7）	12.6（9.5-18.5）	11.0（8.2-13.7）	19.3（15.6-22.3）	
卡马替尼^[59]^	一线	60	68（55.0-79.7）	98（91.1-100.0）	16.6（8.4-22.1）	12.5（8.3-18.0）	21.4（15.2-30.5）	外周水肿、恶心、血肌酐升高、呕吐、呼吸困难、疲乏、食欲减退
	二线及以上	100	44（34.1-54.3）	82（73.1-89.0）	9.7（5.6-13.0）	5.5（4.2-8.1）	16.8（11.6-23.8）	

MET：间质上皮细胞转化因子；NSCLC：非小细胞肺癌；NE：不可评估；ORR：客观缓解率；DCR：疾病控制率；DoR：缓解持续时间；PFS：无进展生存期；OS：总生存期；CI：置信区间；ALT：丙氨酸氨基转移酶；AST：天门冬氨酸氨基转移酶。

赛沃替尼是我国自主研发的MET-TKI，其治疗METex14跳跃突变NSCLC的IIIb期临床研究（NCT04923945）^[53-54]^显示，初治人群ORR为62.1%，中位缓解持续时间（duration of remission, DoR）为12.5个月，中位PFS为13.7个月，中位OS为28.3个月；经治人群ORR为39.2%，中位DoR为11.1个月，中位PFS为11.0个月，中位OS为25.3个月。

谷美替尼为国产新型MET-TKI，全球多中心、单臂II期GLORY研究^[55]^证实其治疗METex14跳跃突变局部晚期或转移性NSCLC的疗效与安全性，总体ORR为66%，初治ORR为71%，经治ORR为60%；中位DoR为8.2个月；总体中位PFS为8.5个月，初治中位PFS为11.7个月，经治中位PFS为7.6个月；总体中位OS为19.4个月，初治中位OS为25.4个月，经治中位OS为16.3个月；基线伴脑转移患者ORR为85%（11/13），其中5例以脑转移灶为靶病灶者均达到颅内部分缓解（partial response, PR），颅内ORR达100%^[56]^。

KUNPENG研究^[57]^为开放标签、多中心、多队列II期研究，评估伯瑞替尼治疗局部晚期或转移性MET异常NSCLC的疗效和安全性。截至2023年8月9日，METex14跳跃突变队列共纳入52例患者，中位随访19.1个月。经盲态独立评审委员会（Blinded Independ Review Committee, BIRC）评估，总体ORR为75.0%，DCR为96.2%，中位DoR为16.5个月，中位PFS为14.3个月，中位OS为20.3个月，3年OS率为35.1%。亚组分析显示，初治患者ORR为77.1%，经治患者ORR为70.6%，伴MET扩增或脑转移者ORR均为100%，肝转移者ORR为66.7%，≥75岁高龄患者ORR为85.7%。

VISION研究^[58]^为特泊替尼的II期研究，纳入METex14跳跃突变局部晚期或转移性NSCLC。经液体或组织活检确认METex14跳跃突变状态，结果显示，总体人群ORR为51.4%，中位DoR为18.0个月，中位PFS为11.2个月，中位OS为19.6个月。

GEOMETRY mono-1研究^[59]^为卡马替尼的II期研究，初治队列ORR为68%，中位DoR为16.6个月，中位PFS为12.5个月，中位OS为21.4个月；经治队列ORR为44%，中位DoR为9.7个月，中位PFS为5.5个月，中位OS为16.8个月。

此外，非TKIs类药物也在积极探索中。埃万妥单抗（Amivantamab）是全球首个获批上市的EGFR/MET双特异性抗体。在CHRYSALIS研究^[60]^的METex14队列中，ORR为33%（31/97, 95%CI: 23%-42%），其中未接受过MET-TKIs患者的ORR为46%（13/28, 95%CI: 28%-66%），接受过MET-TKIs患者的ORR为19%（10/53, 95%CI: 9%-32%），中位DoR为11.2个月（95%CI: 5.3-19.0）。

专家共识5：对于METex14跳跃突变的局部晚期或转移性NSCLC患者，可使用赛沃替尼、谷美替尼、伯瑞替尼、特泊替尼或卡马替尼进行治疗（1A）。

### 3.2 MET扩增NSCLC的治疗

AcSé研究^[61]^、GEOMETRY mono-1研究^[59]^、VISION研究^[62]^结果提示，MET-TKIs可能为MET扩增的晚期NSCLC患者带来获益。2025年6月24日，伯瑞替尼获得NMPA附条件批准用于治疗MET扩增的局部晚期或转移性NSCLC，改变了该领域的治疗格局。

AcSé研究^[61]^显示，克唑替尼单药治疗原发MET扩增（GCN≥6）NSCLC，ORR为16%，中位PFS为3.2个月，中位OS为7.7个月。GEOMETRY mono-1研究^[59]^证实，卡马替尼对原发MET扩增患者有效，且疗效与扩增水平呈正相关，在MET GCN≥10的高扩增患者中，初治患者ORR为40%，经治患者为29%。VISION研究^[62]^队列B评估特泊替尼治疗MET扩增NSCLC，24例患者中一线治疗7例、二线治疗10例、三线治疗7例，独立评审委员会（Independent Review Committee, IRC）评估的全组ORR为41.7%，一线治疗ORR为71.4%（5/7），二线治疗ORR为27.3%（3/11），三线治疗ORR为33.3%（2/6）。KUNPENG研究中的队列2和队列3评价了伯瑞替尼治疗MET扩增NSCLC的疗效^[63]^。截至2024年11月14日，共纳入86例患者（初治56例，经治30例），全组患者ORR为48.8%，中位DoR为12.1个月，中位PFS为7.4个月，中位OS为13.9个月。初治与经治患者的ORR分别为51.8%（29/56）和43.3%（13/30），中位DoR分别为12.1和11.0个月，中位PFS分别为8.3和7.4个月，中位OS分别为15.3和12.0个月。

专家共识6：MET扩增阳性的晚期NSCLC患者可使用伯瑞替尼（2A）、卡马替尼（2B）、特泊替尼（2B）或克唑替尼（2B）进行治疗。

### 3.3 EGFR-TKIs耐药后MET扩增NSCLC的治疗

多项临床研究^[38-42,64-66]^表明，部分接受EGFR-TKIs治疗后继发MET扩增的NSCLC患者，从化疗、免疫治疗中获益有限。EGFR-TKIs联合MET-TKIs是克服此类耐药的潜在治疗策略。

SAVANNAH研究^[64]^是一项II期研究，评估赛沃替尼联合奥希替尼治疗第三代EGFR-TKIs耐药后MET扩增和/或过表达NSCLC患者的疗效。截至2021年8月，ORR为32%，中位DoR为8.3个月，中位PFS为5.3个月。MET高扩增/高过表达[GCN≥10和/或IHC≥90%肿瘤细胞强着色（3+）]亚组获益更大，ORR达49%，中位DoR达9.3个月，中位PFS达7.1个月；而非高扩增/高过表达亚组获益有限，ORR仅9%，中位PFS为2.8个月。SAVANNAH研究最终入组80例MET高扩增/高过表达患者，ORR为56.3%，中位DoR为7.1个月，中位PFS为7.4个月。

SACHI研究^[65]^是一项III期研究，评估赛沃替尼联合奥希替尼对比化疗用于一线EGFR-TKIs进展后伴MET扩增的晚期NSCLC的疗效和安全性。结果显示，联合治疗组在ORR（58% vs 34%）、DCR（89% vs 67% ）方面均显著优于化疗组，两组中位PFS分别为8.2和4.5个月。基于此结果，2025年6月24日，NMPA批准赛沃替尼用于EGFR突变阳性、经EGFR-TKIs治疗后进展的伴MET扩增局部晚期或转移性非鳞NSCLC的治疗。

SCC244-203研究^[39]^为探索谷美替尼联合奥希替尼用于第一、二、三代EGFR-TKIs耐药后MET扩增NSCLC疗效的Ib/II期临床试验。研究结果显示，全组患者ORR达60%，中位PFS为6.9个月，中位OS可达16.9个月。

INSIGHT研究^[40]^头对头对比特泊替尼联合吉非替尼与化疗，证实联合靶向方案可显著改善患者生存获益与应答结局。联合组中位PFS为16.6个月，中位OS为37.3个月，ORR为66.7%，中位DoR为19.9个月，全面优于化疗组（中位PFS为4.2个月，中位OS为13.1个月，ORR为42.9%，中位DoR为2.8个月）。

INSIGHT 2研究^[41]^评估特泊替尼联合奥希替尼用于一线奥希替尼进展后、伴MET扩增的EGFR突变NSCLC，结果显示ORR为50%，中位DoR为8.5个月，不合并其他奥希替尼耐药标志物者预后更佳。

另有一项Ib/II期研究^[42]^显示，卡马替尼联合吉非替尼用于EGFR-TKIs治疗失败后、伴MET扩增或MET蛋白过表达NSCLC患者，II期研究入组患者ORR为29%（29/100），中位DoR为5.6个月；高MET扩增（GCN≥6）亚组患者ORR为47%（17/36）。

KYLIN-1研究^[66]^是一项Ib/II期研究，旨在评估伯瑞替尼联合PLB1004（安达艾替尼）治疗EGFR-TKIs耐药后伴MET扩增/过表达NSCLC患者的有效性和安全性。值得注意的是，研究入组的NGS MET扩增阳性患者，无拷贝数要求。研究结果显示，确认的ORR为50.0%，DCR为89.3%，mPFS为9.9个月。经第一、二代EGFR-TKIs治疗后进展患者，DCR为87.8%，经第三代EGFR-TKIs治疗后进展患者，DCR为89.8%，mPFS为9.6个月。

MET扩增驱动/继发扩增的靶向药物治疗局部晚期或转移性NSCLC的关键临床研究数据见表2^[38-42,59,61-63,65,66]^。

**表 2:** MET扩增驱动/继发扩增的靶向药物治疗局部晚期或转移性NSCLC的关键临床研究数据

药物名称	治疗线数	n	基因扩增水平	ORR (%，95%CI)	DCR (%，95%CI)	中位DoR（月，95%CI）	中位PFS （月，95%CI）	中位OS （月，95%CI）
MET扩增驱动的靶向药物								
克唑替尼^[61]^		25	GCN≥6（FISH）	32	52（4个周期）		3.2（1.9-3.7）	7.7（4.6-15.7）
卡马替尼^[59]^	一线	15	GCN≥10（FISH）	40（16-68）	67（38-88）	7.5（2.6-14.3）	4.2（1.4-6.9）	
	二线及以上	69	GCN≥10（FISH）	29（19-41）	71（59-81）	8.3（4.2-15.4）	4.1（2.9-4.8）	
特泊替尼^[62]^	总体人群	24	GCN≥2.5（NGS）	42（22-63）		NE（2.8-NE）	4.2（1.4-NE）	
	一线	7	GCN≥2.5（NGS）	71（29-96）		NE（2.8-NE）	NE（1.4-NE）	
	二线	10	GCN≥2.5（NGS）	30（7-65）		NE（NE-NE）	NE（1.0-NE）	
	三线	7	GCN≥2.5（NGS）	29（4-71）		NE（3.2-NE）	1.4（0.6-4.5）	
伯瑞替尼^[63]^	总人群	86	GCN≥6（FISH）	48.8（38.3-59.4）	77.9（69.1-86.7）	12.1（6.5-16.8）	7.4（4.7-12.9）	13.9（9.0-16.3）
	一线	56		51.8（38.7-64.9）	80.4（70.0-90.8）	12.1（5.4-16.8）	8.3（4.6-14.7）	15.3（8.9-17.1）
	二线及以上	30		43.3（25.6-61.1）	73.3（57.5-89.2）	11.0（3.7-NE）	7.4（3.7-13.6）	12.0（5.4-18.0）
MET继发扩增的靶向药物								
赛沃替尼+奥希替尼^[38]^	一线EGFR-TKIs （第一、二、三代）经治	193		32（26-39）		8.3（6.9-9.7）	5.3（4.2-5.8）	
	高扩增/表达人群	108	GCN≥10（FISH）	49（39-59）		9.3（7.6-10.6）	7.1（5.8-8.0）	
	低扩增/表达人群	77	GCN<10（FISH）	9（4-18）		6.9（4.1-16.9）	2.8（2.6-4.3）	
赛沃替尼+奥希替尼^[65]^	一线奥希替尼 经治	106	第一、二代EGFR-TKIs经治：GCN≥5或MET/CEP7≥2.0；第三代EGFR-TKIs经治：GCN≥10（FISH）	58（49-68）	89（81-84）	8.4（5.9-11.1）	8.2（6.9-11.2）	
谷美替尼+奥希替尼^[39]^	EGFR-TKIs（第一、二、三代）经治	30	GCN≥4或MET/CEP7≥1.8（FISH）	60.0（40.6-77.3）	90.0（73.5-97.9）	5.8（3.9-12.7）	6.9（3.9-8.9）	16.9（11.1-NE）
特泊替尼+吉非替尼^[40]^	EGFR-TKIs（第一、二代）经治	12	GCN≥5或MET/CEP7≥2 （FISH）	66.7（39.1-87.7）		19.9（7.0-NE）	16.6（8.3-22.1）	37.3（21.1-52.1）
特泊替尼+ 奥希替尼^[41]^	一线奥希替尼 经治	98	GCN≥5或MET/CEP7≥2或血浆GCN≥2.3 （FISH、NGS）	50.0（39.7-60.3）		8.5（6.1-NE）	5.6（4.2-8.1）	17.8（11.1-NE）
卡马替尼+吉非替尼^[42]^	EGFR-TKIs （第一、二代） 经治	27	GCN≥5和/或MET/CEP7≥2	5.6				
伯瑞替尼+安达艾替尼^[66]^	EGFR-TKIs （第一、二、三代）经治	56	GCN≥5和/或MET/CEP7≥2或GCN<5且MET/CEP7<2但NGS阳性 （FISH、NGS）	50.0（36.3-63.7）		8.3（2.8-NA）	9.9（5.5-NA）	

EGFR-TKIs：表皮生长因子受体-酪氨酸激酶抑制剂；NGS：下一代测序；FISH：荧光原位杂交；GCN：基因拷贝数；CEP7：7号染色体着丝粒；NA：无法获得。

专家共识7：对于EGFR-TKIs耐药后出现MET扩增阳性的晚期NSCLC患者，可使用奥希替尼联合赛沃替尼（1B），或EGFR-TKIs联合谷美替尼、伯瑞替尼、特泊替尼、卡马替尼（2B）进行治疗。

### 3.4 EGFR-TKIs耐药后MET蛋白过表达NSCLC的治疗 INSIGHT^[49]^及TATTON^[50]^研究结果表明，EGFR-TKIs耐药后，IHC≥50%肿瘤细胞强着色（3+）的NSCLC患者可能从EGFR-TKIs与MET-TKIs联合治疗中获益，而IHC≥50%肿瘤细胞中等着色（2+）者获益有限。SAVANNAH研究^[64]^进一步证实，奥希替尼耐药后，IHC≥90%肿瘤细胞强阳性（3+）患者接受奥希替尼联合赛沃替尼治疗可获益。此外，非TKIs类药物Teliso-V也在MET异常NSCLC治疗中取得进展。一项Ib期研究^[67]^显示，Teliso-V联合厄洛替尼用于经治c-Met阳性NSCLC，在36例可评估患者中，ORR为32.1%（9/28），中位PFS为5.9个月；MET高表达（H评分≥225）者ORR为52.6%（8/15），抗肿瘤活性良好、安全性可控。CHRYSALIS研究^[68]^探索了埃万妥单抗联合兰泽替尼用于奥希替尼耐药、EGFR突变阳性、IHC EGFR/MET表达H评分≥400的晚期NSCLC，10例患者中9例获得PR，中位DoR为9.7个月，中位PFS为12.5个月。

专家共识8：对于EGFR-TKIs耐药后MET蛋白过表达[IHC≥50%肿瘤细胞强着色（3+）]的晚期NSCLC患者，可使用奥希替尼联合赛沃替尼，或EGFR-TKIs联合谷美替尼、伯瑞替尼、特泊替尼、卡马替尼进行治疗（2B）。

### 3.5 驱动基因阴性MET蛋白过表达NSCLC的治疗

谷美替尼两项研究（NCT03457532、NCT04270591）汇总分析^[51]^显示，谷美替尼单药治疗驱动基因阴性、MET过表达（IHC≥50%、3+）NSCLC，ORR为37.5%（12/32, 95%CI: 21.1%-56.3%），中位DoR为10.3个月（95%CI: 2.8-NE），中位PFS为6.9个月（95%CI: 3.6-9.7），中位OS为17.0个月（95%CI: 10.3-NE）。Gleam研究^[69]^是一项II期试验，纳入局部晚期/转移性驱动基因阴性MET过表达[IHC≥50%肿瘤细胞强着色（3+）]NSCLC，这些患者既往接受免疫及含铂双药化疗后进展或不耐受，给予谷美替尼（300 mg qd）联合白蛋白结合型多西他赛（50或75 mg/m²，q3w）治疗。结果显示，23例患者的ORR为34.8%（8/23），DCR为95.7%（21/23）。其中IHC 3+亚组（n=5）ORR为60%（3/5），DCR为100%；IHC 2+亚组（n=18）ORR为27.8%（5/18），DCR为94.4%。伯瑞替尼I期研究^[70]^显示，单药治疗MET蛋白过表达[IHC≥50%肿瘤细胞强着色（3+）]NSCLC的ORR为35.7%（n=14）。在LUMINOSITY研究^[71]^中，Teliso-V单药二线治疗EGFR野生型、MET蛋白过表达[定义为IHC≥25%肿瘤细胞强着色（3+）；高表达：IHC≥50%肿瘤细胞强着色（3+），中表达：IHC 25%-50%肿瘤细胞强着色（3+）]的晚期非鳞NSCLC，全组ORR为28.6%（46/161），DCR为59.0%，中位DoR为8.3个月，中位PFS为5.7个月，中位OS为14.5个月。c-Met高表达组（n=78）ORR为34.6%，中位PFS为5.5个月，中位OS为14.6个月；中表达组（n=83）ORR为22.9%，中位PFS为6.0个月，中位OS为14.2个月。基于LUMINOSITY结果，美国FDA于2025年5月14日加速批准Teliso-V用于经治EGFR野生型c-MET高表达[IHC≥50%肿瘤细胞强着色（3+）]非鳞NSCLC，但该药尚未在中国NMPA获批。

专家共识9：对于既往经治的驱动基因阴性MET蛋白过表达患者，可尝试使用谷美替尼单药、谷美替尼联合化疗或伯瑞替尼单药治疗（2B）。

## 4 MET-TKIs的临床合理用药

MET异常，尤其是METex14跳跃突变，在NSCLC中多见于高龄患者，常合并心脑血管疾病、糖尿病及肝肾功能减退等基础疾病，导致药物代谢及耐受存在个体差异。更重要的是，多病共存状态常需多药联合治疗，显著增加复杂药物相互作用（drug-drug interaction, DDI）的风险。因此，在保障疗效的前提下，主动监测不良反应、及时干预并进行个体化综合评估与动态管理，是实现MET-TKIs安全用药的核心（表3）。

**表 3:** MET-TKIs药学特性及特殊人群用药管理

项目		赛沃替尼	谷美替尼	伯瑞替尼	特泊替尼	卡马替尼
主要代谢途径		肝脏代谢，主要依赖CYP450酶系统	磺酰基水解，肝代谢途径<5%	肝脏代谢，主要依赖CYP450酶系统	肝脏代谢，主要依赖CYP450酶系统	肝脏代谢，主要依赖CYP450酶系统
肝脏相关不良反应	≥3级ALT升高	8.9%	1.0%	6.5%	4.8%	7.0%
	≥3级AST升高	8.3%	1.3%	5.1%	3.5%	3.5%
肾脏相关不良反应	≥3级血肌酐升高	-	0.0%	0.0%	1.0%	0.3%
心脏相关不良反应	≥3级心电图QT间期延长	1.0%	1.7%	0.7%	-	-
肝功能不全患者剂量调整	轻度	无需调整	无需调整	无需调整	无需调整	无需调整
	中度	尚不明确，慎用	尚不明确，慎用	尚不明确，慎用	无需调整	无需调整
	重度	尚不明确，慎用	尚不明确，慎用	尚不明确，慎用	尚不明确	无需调整
肾功能不全患者剂量调整	轻度	无需调整	无需调整	无需调整	无需调整	无需调整
	中度	无需调整	无需调整	无需调整	无需调整	无需调整
	重度	尚不明确，慎用	尚不明确，慎用	尚不明确，慎用	尚不明确	尚不明确，慎用
药物相互作用	强CYP3A4诱导剂/抑制剂	避免	无限制	避免	避免	避免
	P-gp底物	慎用	无限制	无限制	慎用	慎用

CYP450：细胞色素P450。

### 4.1 MET-TKIs常见不良反应

MET-TKIs总体安全性良好，不同药物的不良反应谱及发生率存在差异。需重点关注的不良反应包括外周水肿、肝毒性、胃肠道反应、间质性肺炎及QT间期延长等，多数为1-2级，经积极处理可完全缓解。外周水肿是最具特征性的不良事件，发生率为40%-70%，轻中度可通过限盐、抬高患肢、穿弹力袜等缓解，必要时短期使用利尿剂并监测电解质。肝毒性多表现为转氨酶及胆红素升高，常于用药后2-8周出现，建议治疗初期每2-4周监测肝功能，根据通用不良事件术语标准（Common Terminology Criteria for Adverse Events, CTCAE），胆红素升高≥2级或丙氨酸氨基转移酶（alanine aminotransferase, ALT）/天门冬氨酸氨基转移酶（aspartate aminotransferase, AST）升高≥3级时应暂停给药。胃肠道反应（恶心、呕吐、腹泻）多为轻中度，必要时可使用5-羟色胺-3（5-hydroxytryptamine-3, 5-HT3）受体拮抗剂等止吐药及洛哌丁胺等止泻药对症处理。间质性肺炎是严重不良事件，发生率虽低但需高度警惕，一旦出现新发或加重的呼吸困难、干咳、发热等症状，应立即停药，影像学确诊后及时启动糖皮质激素治疗。QT间期延长发生率为1%-5%，在存在心脏基础疾病或联用延长QTc间期药物的患者中风险增高，用药前及治疗期间应定期复查心电图，QTc间期>500 ms或较基线延长>60 ms时应减量或停药。

### 4.2 肝肾功能不全患者的用药考量

对于合并肝肾功能不全的MET异常NSCLC患者，应基于药代动力学特征审慎选药，优先考虑肝肾相关不良反应风险较低的药物。理想情况下，应优先选择在该类人群中已有明确研究数据及剂量调整建议的药物。若选择尚无特定人群安全性数据但说明书未禁用的药物，则必须依据其清除途径，如主要经肝脏细胞色素P450（cytochrome P450, CYP450）酶代谢或经肾脏原型排泄的比例、蛋白结合率等，评估潜在风险，并考虑可能需要调整起始剂量。治疗期间需加强对ALT、AST、总胆红素等肝功能指标及血清肌酐、肾小球滤过率等肾功能指标的监测频度，及时发现和处理药物相关的肝肾功能损伤。

### 4.3 存在心脏基础疾病患者的用药风险管理

存在心脏基础疾病是MET-TKIs治疗中需重点评估的风险因素。药物选择前应对患者进行心电图、心脏超声等检查，评估心脏功能。选药时应平衡抗肿瘤疗效与潜在心脏毒性（如QT间期延长、心动过缓、左心室射血分数下降等），倾向于选择心脏毒性报告较少或程度较轻的药物。治疗期间需定期复查心电图等心脏功能检查，密切关注相关症状，以便早期识别和处理心脏不良事件。

### 4.4 DDI的评估与管理

MET-TKIs的药代动力学过程易受合并用药影响，主要涉及CYP450酶系，尤其是CYP3A4。与伊曲康唑、克拉霉素、利托那韦等CYP3A4强效抑制剂联用，可能显著抑制其代谢，导致血药浓度升高，增加不良反应风险；反之，与利福平、卡马西平、苯妥英钠等强效诱导剂联用，则会加速代谢，降低血药浓度，可能影响疗效。此外，部分MET-TKIs与P-糖蛋白（P-glycoprotein, P-gp）等药物转运蛋白也存在相互作用。因此，临床医师和药师在处方前应全面审查患者的全部用药清单。一旦发现具有临床意义的DDI，应根据药品说明书、权威数据库及指南建议，采取调整MET-TKIs剂量、更换合并用药或加强特定毒性监测等干预措施，确保用药安全。

专家共识10：MET-TKIs的临床合理用药需综合评估患者肝肾功能、心脏等基础疾病及合并用药情况，进行个体化管理（2A）。

## 5 展望

### 5.1 MET扩增/过表达判读阈值

目前，FISH和NGS等MET异常检测方法的判读阈值尚未统一。随着分子机制研究的深入，建立标准化判读体系、提高不同机构间检测结果的可比性和重复性将成为可能。新一代NGS凭借更高测序深度与更广基因组覆盖，可更灵敏、特异地检出MET扩增；而循环肿瘤DNA（circulating tumor DNA, ctDNA）液体活检的应用，则为MET扩增状态的动态监测提供了非侵入性新途径，可实时追踪肿瘤演变过程中MET扩增状态的变化。但仅检测扩增无法全面评估肿瘤MET依赖性，未来研究应考虑整合MET蛋白表达、基因突变状态等多维度生物标志物，以更精准地预测靶向治疗疗效、减少无效治疗及其带来的不良反应。随着临床数据积累，MET扩增/过表达与预后、疗效的关联性将进一步明确，为制定最优判读阈值提供依据。通过系统评估获益-风险，有望建立MET异常分级体系，推动个体化精准治疗。

### 5.2 MET扩增NSCLC患者的治疗

卡马替尼、特泊替尼等MET-TKIs已在MET扩增NSCLC中展现初步疗效。伯瑞替尼于2025年6月获NMPA附条件批准用于MET扩增NSCLC，相关研究成果同步在国际学术会议及权威期刊发表^[63]^，进一步确立MET扩增作为独立驱动基因的地位，为同类药物研发提供重要参考。

未来可通过优化给药方案、研发高选择性MET-TKIs，针对不同扩增水平实施个体化治疗。MET扩增常与EGFR、ALK等驱动变异共存，MET-TKIs联合对应靶向药物有望实现协同增效。当前研究观察到MET扩增水平与MET-TKIs疗效呈正相关，未来基于扩增水平的分层治疗将进一步提升精准度。治疗期间应动态监测MET扩增状态、疗效及耐药变异，及时调整治疗策略。

### 5.3 MET蛋白过表达治疗

MET蛋白过表达既可与其他驱动基因（如EGFR突变）共存并介导靶向治疗耐药，也可作为独立致癌驱动事件存在。针对不同类型的MET过表达患者，ADC等新型药物的应用正带来重要突破。在EGFR-TKIs耐药后MET过表达领域，ADC已展现出良好抗肿瘤活性与协同潜力。RC108联合第三代EGFR-TKIs的Ib/II期研究^[72]^显示，在18例≥10%肿瘤细胞膜IHC 1+/2+/3+且≤20%肿瘤细胞强着色（3+）的患者中，ORR为50.0%，DCR为83.3%。在驱动基因阴性MET蛋白过表达领域，除已获美国FDA批准的Teliso-V外，多个c-MET ADC已进入临床研究。ABBV-400采用拓扑异构酶I抑制剂作为载荷，I期研究显示在含铂化疗及EGFR-TKIs经治NSCLC中ORR为63%（26/41），且缓解率不依赖c-Met表达水平^[73]^；SHR-1826的I期研究^[74]^显示，既往多线治疗NSCLC的ORR为39.7%（23/58），DCR达94.8%，疗效覆盖不同c-MET表达水平。ABBV-400、SHR-1826、RC108等新一代ADC在临床研究中展现出令人鼓舞的抗肿瘤活性，为拓展MET过表达NSCLC的治疗路径提供了重要方向。未来需继续积累循证医学证据，通过大型确证性研究明确ADC的临床定位和应用时机，同时推动MET IHC判读标准的统一与优化，实现精准分层与全程管理。

### 5.4 MET融合治疗

MET融合阳性NSCLC的靶向治疗以MET-TKIs为主。有研究^[75]^不仅验证了已知融合伴侣，还发现EPHB4、THAP5、TNPO3、DST等新型融合类型。其中，EPHB4-MET融合患者常规MET检测（IHC、FISH），METex14均为阴性，但对MET-TKIs持续应答，提示其为潜在新靶点。

一项I期研究数据^[75]^显示，12例MET融合患者接受MET-TKIs治疗，6例达PR、2例稳定、4例进展，反映出不同融合亚型的疗效异质性。研究表明EPHB4-MET融合可介导EGFR/ALK-TKIs耐药；MET-DST融合可与METex14跳跃突变共存。此外，治疗过程中会出现继发性耐药，患者可能在获得性耐药后携带MET D1228H/N或D1246N等突变，影响治疗效果，因此需不断探索新的MET-TKIs和联合方案。Tivantinib在肿瘤类器官模型中表现出对携带MET D1228N突变患者的较好抑制效果。在SPARTA-II临床研究^[76]^中，通过DNA全外显子或RNA全转录组测序发现14例MET融合阳性患者（其中7例NSCLC），接受伯瑞替尼治疗后，ORR达50%（7/14），DCR为79%（11/14）；在7例NSCLC中，ORR为57%（4/7），DCR为85.7%（6/7），其中有2例治疗时间超过3年仍未进展。

### 5.5 MET异常的其他类型

除上述4种主要变异类型外，MET基因异常还包括TKD突变和激酶结构域复制（kinase domain duplication, KDD）等特殊类型。MET TKD突变常导致MET-TKIs耐药，目前尚无标准治疗方案。KDD作为受体酪氨酸激酶活化的新机制，在EGFR和MET等基因中均有发现。一项临床数据分析^[77]^显示，14例MET KDD患者中，NSCLC 6例，其他肿瘤8例，部分患者对MET-TKIs显示一定疗效，仍需进一步探索有效治疗策略^[78]^。

### 5.6 MET异常NSCLC患者靶向治疗进展后的治疗

MET-TKIs耐药机制复杂，主要包括靶点依赖性耐药（MET TKD突变，削弱药物结合）、旁路激活耐药[Erb-b2受体酪氨酸激酶（Erb-b2 receptor tyrosine kinases, ERBB）家族扩增导致旁路和/或下游信号激活]及混合型耐药等。针对靶点依赖性耐药，I与II型MET-TKIs间切换可能产生部分疗效。卡马替尼对克唑替尼耐药患者显示一定疗效（DCR 80%）^[79]^。埃万妥单抗对MET-TKIs失败患者具有抗肿瘤活性^[74]^；而旁路耐药的有效治疗方案仍待深入研究。国内临床调研^[6]^显示，对于METex14跳跃突变NSCLC患者，无论初治还是经治，超过50%的中国医生首选MET-TKIs单药治疗；对于合并MET扩增的EGFR突变NSCLC患者，在EGFR-TKIs耐药后，医生更倾向于选择MET-TKIs联合EGFR-TKIs双靶治疗。

### 5.7 可手术MET异常NSCLC的治疗

在MET-TKIs应用于MET异常早期NSCLC围手术期治疗方面，仍缺少高证据级别的前瞻性临床研究结果。Geometry-N（NCT04926831）研究入组IB-IIIA期（含部分N2及IIIB期）可切除NSCLC，平行纳入METex14跳跃突变及MET扩增两个队列，采用卡马替尼术前8周单药新辅助联合术后最长3年辅助治疗的模式，该研究已提前终止，无相关结果发表。NCT07156604研究聚焦可切除IIA-IIIB（N2）期METex14跳跃突变NSCLC，评估伯瑞替尼新辅助治疗8周后手术的疗效与安全性，试验于2025年9月启动。一项由上海市肺科医院发起的前瞻性、单中心II期研究（NCT06644313），旨在评估伯瑞替尼±含铂双药化疗4个周期用于IIIA-IIIB（N2）期MET异常NSCLC新辅助治疗的疗效，已在2025年世界肺癌大会（World Conference of Lung Cancer, WCLC）上公布了3例患者疗效。ARM研究（NCT03088930）针对可手术切除且ALK、ROS1或MET基因阳性NSCLC，评估克唑替尼新辅助治疗效果，研究于2021年1月完成，但无公开数据发表。NCT05800340研究探索特瑞普利单抗联合化疗在包括METex14跳跃突变及MET扩增在内罕见驱动基因变异局部晚期NSCLC新辅助及辅助治疗中的应用，正在进行中。此外，LEADER（NCT04712877）等多个围手术期伞式筛选平台已将METex14跳跃突变及MET扩增纳入前瞻性靶向治疗临床研究，筛选后的阳性患者接受MET-TKIs新辅助治疗，相关研究均在进行中。

个案报道^[80-84]^为MET-TKIs的临床探索提供了实践依据。Rotow等^[80]^报道1例亚裔女性，右上肺肿物直径15 cm，伴纵隔淋巴结肿大，基因检测示METex14跳跃突变及MET扩增，接受克唑替尼新辅助治疗8个月后手术，术后病理达完全缓解，并继续克唑替尼辅助治疗，耐受良好，术后6个月随访无复发。目前已有多个MET-TKIs新辅助治疗成功案例^[80-83]^，患者接受术前4-10周赛沃替尼治疗后成功缩瘤降期，得以根治性切除。术后辅助策略各异：Zhang等^[81]^和Deng等^[82]^报道持续使用赛沃替尼辅助治疗；Tian等^[83]^报道降期后依据术后分期决定不进行MET-TKIs辅助治疗，仅观察随诊；Fu等^[84]^报道术后采用标准培美曲塞联合卡铂辅助化疗。3例METex14跳跃突变IIIA-IIIB期NSCLC患者接受伯瑞替尼治疗4个月后手术，术后病理证实1例病理完全缓解，1例主要病理缓解，1例非主要病理缓解，表明MET-TKIs作为新辅助治疗可为该类患者带来获益。现有临床个案提示，对于高龄、拒绝化疗或存在化疗禁忌的晚期NSCLC患者，可尝试探索性使用MET-TKIs。对于携带METex14跳跃突变的早期NSCLC，亟需前瞻性多中心临床研究验证MET-TKIs在围手术期治疗中的有效性与安全性。

## 6 总结

总之，MET异常是NSCLC患者中常见的驱动基因变异。中国NMPA已批准多种MET-TKIs用于治疗METex14跳跃突变和MET扩增阳性的晚期NSCLC患者，而针对MET蛋白过表达人群的靶向治疗策略仍在探索中。本专家共识参考了国际权威诊疗指南和最新研究进展，并结合中国国情制定。鉴于临床实践中患者存在较大个体差异，需根据具体情况决定每位患者的治疗策略。本专家共识仅供参考，不具有法律效力。

**MET异常非小细胞肺癌诊疗中国专家共识（2026年版）**

**编写委员会**

**组长：**陈军（天津医科大学总医院），韩宝惠（上海交通大学医学院附属胸科医院），胡毅（中国人民解放军总医院），胡坚（浙江大学医学院附属第一医院），石远凯（航天中心医院；国家癌症中心 国家肿瘤临床医学研究中心 中国医学科学院北京协和医学院肿瘤医院）

**执笔专家**（按姓氏汉语拼音首字母排序）：白俊（陕西省人民医院），毕清（云南省肿瘤医院），常香云（天津医科大学总医院），车国卫（四川大学华西医院），陈钢（天津医科大学总医院），陈骏（大连医科大学附属第二医院），陈鹏（天津医科大学肿瘤医院），陈晓（吉林大学附属第一医院），褚倩（华中科技大学同济医学院附属同济医院），董桂兰（唐山市人民医院），董明（天津医科大学总医院），董文（海南省肿瘤医院），董晓荣（华中科技大学同济医学院附属协和医院），段建春（中国医学科学院肿瘤医院），范海洋（中山大学第七医院），范云（浙江省肿瘤医院），冯慧晶（山西白求恩医院），葛洋（首都医科大学附属北京朝阳医院），郭卉（西安交通大学第二附属医院），郭克锋（黄河三门峡医院），郭人花（江苏省人民医院），郭伟（山西省肿瘤医院），郭占林（内蒙古医科大学第一附属医院），韩琤波（中国医科大学附属盛京医院），韩颖（天津医科大学肿瘤医院），韩昱晨（上海交通大学医学院附属胸科医院），何志锋（温州医科大学附属第一医院），胡兴胜（中国医学科学院肿瘤医院），黄广慧（山东第一医科大学附属省立医院），黄岩（中山大学附属肿瘤医院），黄韵坚（福建省肿瘤医院），康世荣（内蒙古医科大学附属医院），雷会宁（金昌市中心医院），李琳（北京医院），李蓉（金昌市中心医院），李书军（河北医科大学第二医院），李昕（天津医科大学总医院），李燕巍（天津医科大学肿瘤医院），李咏生（重庆大学附属肿瘤医院），李源（金昌市中心医院），李玉苹（温州医科大学附属第一医院），李昀（中山大学附属第七医院），李正国（甘肃省武威肿瘤医院），李志杰（潍坊市第二人民医院），梁华刚（秦皇岛市第一医院），刘春玲（新疆医科大学附属肿瘤医院），刘东颖（天津医科大学肿瘤医院），刘红雨（天津医科大学总医院），刘京豪（天津医科大学总医院），刘明辉（天津医科大学总医院），刘仁旺（天津医科大学总医院），刘勇（空军军医大学第二附属医院），龙浩（中山大学附属肿瘤医院），龙玥（天津医科大学总医院），孟凡路（天津医科大学总医院），孟庆威（哈尔滨医科大学附属肿瘤医院），孟祥姣（山东第一医科大学附属肿瘤医院），潘跃银（安徽省肿瘤医院），商琰红（河北大学附属医院），申思宁（河南省肿瘤医院），史丽霞（天津市海河医院），宋作庆（天津医科大学总医院），孙龙华（南昌大学第一附属医院），唐可京（中山大学附属第一医院），田子强（河北医科大学第四医院），王海永（山东省肿瘤医院），王雷（河北医科大学第四医院），王启鸣（河南省肿瘤医院），王晟广（天津医科大学肿瘤医院），王勇杰（青岛大学附属医院），王永生（四川大学华西医院），王玉栋（河北医科大学第四医院），王宇飞（内蒙古医科大学附属医院），王章桂（航天中心医院），韦森（天津医科大学总医院），邬麟（湖南省肿瘤医院），吴芳（中南大学湘雅二医院），吴凤英（同济大学附属上海市肺科医院），吴君（天津医科大学总医院），项轶（上海交通大学医学院附属瑞金医院），肖奎（中南大学湘雅二医院），徐嵩（天津医科大学总医院），徐松涛（上海市老年医学中心），徐燕（北京协和医院），杨立伟（河北医科大学第四医院），杨哲（山东省立医院），姚文秀（四川省肿瘤医院），姚颐（武汉大学人民医院），姚煜（西安交通大学第一附属医院），于雁（哈尔滨医科大学附属肿瘤医院），虞永峰（上海交通大学医学院附属胸科医院），翟静（天津医科大学总医院），张广健（西安交通大学第一附属医院），张洪兵（天津医科大学总医院），张红梅（空军军医大学西京医院），张军航（中山大学附属第七医院），张同梅（首都医科大学附属北京胸科医院），张文学（天津医科大学总医院），张新伟（天津医科大学肿瘤医院），赵洪林（天津医科大学总医院），赵明芳（中国医科大学附属第一医院），赵荣志（天津医科大学总医院），周承志（广州医科大学附属第一医院），周斐（同济大学附属东方医院），周建娅（浙江大学医学院附属第一医院），周韶璋（广西医科大学附属肿瘤医院），周晓燕（复旦大学附属肿瘤医院），朱冰（重庆大学附属肿瘤医院），朱大兴（四川大学华西医院），朱豪华（航天中心医院）

**审核专家：**周清华（四川大学华西医院），支修益（首都医科大学宣武医院），何建行（广州医科大学附属第一医院），高树庚（中国医学科学院肿瘤医院），刘伦旭（四川大学华西医院），刘宏旭（辽宁省肿瘤医院）
